# Microbial Response to Coastal-Offshore Gradients in Taiwan Straits: Community Metabolism and Total Prokaryotic Abundance as Potential Proxies

**DOI:** 10.1007/s00248-022-02031-7

**Published:** 2022-05-17

**Authors:** Lingling Wan, Gabriella Caruso, Xiuyun Cao, Chunlei Song, Giovanna Maimone, Alessandro Ciro Rappazzo, Pasqualina Laganà, Yiyong Zhou

**Affiliations:** 1 Institute of Hydrobiology, Chinese Academy of Sciences (CAS-IHB), Wuhan, People’s Republic of China; 2 Institute of Polar Sciences, National Research Council (CNR-ISP), Messina, Italy; 3 BIOMORF Department, University of Messina, Messina, Italy

**Keywords:** Coastal-offshore gradients, Prokaryotic abundance, Carbon substrate utilization patterns, Microbial response, Taiwan Straits

## Abstract

Located between the South and the East China Sea, the Taiwan Straits (TWS) are a marine shelf-channel area, with unique hydrological and geomorphological features affected by rivers inflow and with recent algal blooms with red tide events. This study aimed at assessing microbial distribution and function and their modulation in response to environmental gradients. Surface (0.5 m) water samples from 16 stations along five north to south transects were collected; total prokaryotic abundance by epifluorescence microscope and carbon substrate utilization patterns by Biolog Ecoplates were estimated. Spatially, a patchy microbial distribution was found, with the highest microbial metabolic levels and prokaryotic abundance in the TWS area between Minjiang River estuary and Pingtan Island, and progressive decreases towards offshore stations. Complex carbon sources and carbohydrates were preferentially metabolized. This study provides a snapshot of the microbial abundance and activity in TWS as a model site of aquatic ecosystems impacted from land inputs; obtained data highlights that microbial metabolism is more sensitive than abundance to environmental changes.

**Supplementary Information:**

The online version contains supplementary material available at 10.1007/s00248-022-02031-7.

## Introduction

In aquatic ecosystems, heterotrophic microorganisms including Bacteria and Archaea are recognized to be the major drivers of organic matter turnover and nutrient regeneration; in spite of their small size, these components play a key role in the ocean biogeochemistry [1, 2]. Bacterial growth depends on the availability of organic substrates that can be synthesized during the photosynthetic process such as “autochthonous” organic matter inputs or that enter the marine environment via riverine or continental inputs (such as in the case of “allochthonous” organic matter inputs). In turn, the quantity and quality of the polymers are affected by the decomposition processes performed by the heterotrophs; organic polymers such as proteins, lipids, carbohydrates, and organic phosphates are a very heterogenous matrix and the use of substrates with molecular weight higher than 600 daltons by the microbial community is possible only after their hydrolysis through specific enzymes synthesized by microrganisms themselves [3]. Through microbial metabolism, the organic matter is decomposed into smaller and simple compounds and finally mineralized to carbon dioxide; this process contributes to the recycling of nitrogen and phosphorus needed for primary production and heterotrophic prokaryotic production [4, 5].

Organic matter decomposition and mineralization have significant implications on biogeochemical cycles and on the metabolic functioning of the microbial community, hence contributing to the regulation of matter and energy fluxes through the trophic web [4]. In this context, quali- and quantitative observations on the microbial abundance and function are needed to evaluate the role of microorganisms to organic matter turnover [6, 7]. Since the degradation of organic matter can be dependent upon factors such as substrate type, availability, and community nutrient demands, several physical, chemical, and biological factors can influence microbial metabolism and the enzyme patterns of aquatic microorganisms [8–10].

Marine ecosystems are characterized by diversified physical–chemical and biological variables; to this regard, compared with the open oceans, coastal environments are characterized by high nutrient concentrations and enhanced microbial biomass and productive processes as a result of water enrichment by terrestrial runoff and global climate change [11, 12]. The Taiwan Straits (TWS) are a shelf-channel located between the South and the East China Sea, with unique hydrological and geomorphological features [13] affected by rivers inflow and with recent algal blooms with red tide events. The hydrological patterns of TWS may be directly regulated by Kuroshio Current, South China Sea Warm Current, and China Coastal Current, where the mean water depth is about 60 m [14–17] and the active upwellings of TWS are strongly influenced by the monsoon and terrain uplift [18]. The algal distributions in waters around Taiwan are also affected by the hydrographic features driven by monsoon [19, 20] and the diversities of microeukaryotes show high relationships with the water mass-driven geographic distance and environmental conditions [21]. Hence, the complex environmental heterogeneity makes TWS an ideal system to investigate the diversity and functions of the microbial communities.

Based on the well-known responsiveness of microbial communities to environmental gradients, such as those related to temperature and salinity, availability of both organic matter and inorganic nutrients, and its large spatial and temporal flexibility [2, 22], changes in organic matter supply, or more generally in environmental conditions, as well as in microbial community composition across spatiotemporal scales may be reflected in the metabolic patterns of the microbial community. Consequently, coastal to offshore gradients in terms of organic matter content and nutrient concentrations are expected to affect microbial distribution and metabolic patterns.

To date, the TWS have been scarcely characterized from a microbiological point of view and very few information on the structure and function of microbial community inhabiting this ecosystem is available apart from recent studies [11, 12]. To fill this gap, a multidisciplinary oceanographic cruise was organized within the International program “Mechanisms of red tides and hypoxia as ecological marine disasters and technologies for its early warning along the Belt and Road Countries,” funded by the National Key Research and Development Program of China.

This study aimed at providing a snapshot of the abundance and functional diversity of the microbial communities, focusing on the distribution patterns of their total prokaryotes and the metabolic potential, and at relating them to the environmental characteristics of the TWS. Insights on the suitability of both parameters as potential proxies of microbial response to environmental gradients from coast to offshore were the ultimate goal of this investigation.

## Material and Methods

### Sampling Design and Assayed Parameters

**Fig. 1:**
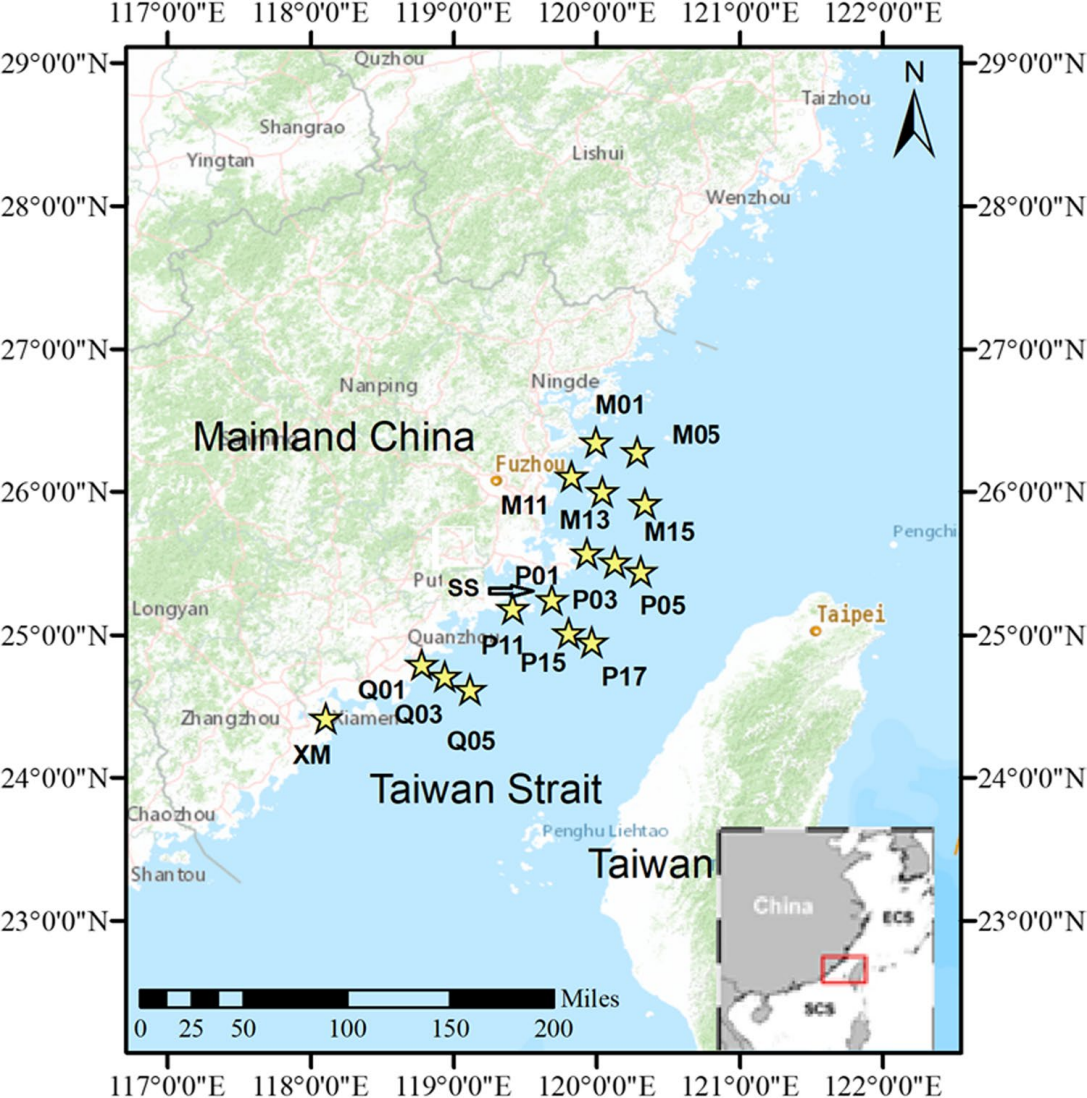
Location of the sampling sites in Taiwan Straits

In May 2019, surface (0.5 m) water samples from 16 stations along five north to south transects were collected on board the R/V China Marine Surveillance 203 (Fig. 1). The main physical–chemical parameters, i.e., temperature, conductivity, salinity, pH, and turbidity, as well as the concentration of total dissolved solids (TDS), chlorophyll-*a*, and blue-green algae-phycoerythrin (BGA-PE) were measured using a CTD probe and after filtration of water volumes through Whatman glass fiber filters, respectively, according to the standard analytical procedures used for the determination of these parameters. The carbon substrate utilization patterns and the total prokaryotic abundance were estimated.

### Microbial Community Metabolism

Microbial community metabolism was studied by analysis of the carbon substrate utilization patterns [23–25]. According to the standard procedure of the CNR-ISP laboratory, Biolog Ecoplates (Rigel Life Sciences, Rome, Italy) were used to determine the differences of the metabolic potentials of microbial assemblages hosted in the samples. Each plate is a 96-well microtiter plate, containing 31 carbon sources and a control in triplicate together with the redox dye tetrazolium violet. Biolog plates were inoculated with 150 μl of sample and incubated at 22 °C in the dark under aerobic conditions. The oxidation of the carbon sources into formazan was quantified by absorbance measurements at 590 nm using a microplate-reader spectrophotometer (MICROTITER ELX-808, Bio Whittaker, Inc.) equipped with an Automatic Microplate Reader and the specific software (WIN KQCL) for data processing. The optical density (OD) of the reaction product was measured at time zero (T0, namely immediately after inoculation), and then every 7 days until 21 days of incubation. The color development for each plate was expressed in terms of the averaged substrate color development (ASCD), which was calculated using the formula *ASCD* = *Σ* [(*R* − *C*)/31], where *R* is the averaged absorbance of the three wells with substrate and *C* is the averaged absorbance of the control wells (without substrate) (according to [24]). The absorbance percentages of each substrate were determined in agreement with Sala et al. [25], setting a value of 2% absorbance of the total absorbance measured per plate as a threshold for substrate utilization. The carbon substrates were grouped into the following six guilds: complex carbon sources (polymers), carbohydrates, phosphate carbon sources, carboxylic and acetic acids, amino acids, and amines.

### Total Prokaryotic Abundance

Water samples for prokaryotic abundance counts were collected in sterile 50-ml-polyethylene tubes, immediately fixed with filter-sterilized formaldehyde (2%, final concentration) and stored at 4 °C in the dark during the transfer in Italy until lab processing. Aliquots of 1 ml were filtered through polycarbonate black membranes (porosity 0.22 µm; GE Water & Process Technologies, USA) and stained for 10–20 min with DAPI (4′6-diamidino-2-phenylindole, final concentration 10 μg ml^−1^ by Sigma-Aldrich, Merck Life Sciences, Milan, Italy) [26]. The prokaryotic cells were quantified by an AXIOPLAN 2 Imaging microscope (Zeiss, Milan, Italy), equipped with a HBO 100 W lamp, filter sets G365 excitation filter, FT395 chromatic beam splitter, LP420 barrier filter, an AXIOCAMHR digital camera, and an AXIOVISION 3.1 software. Cell counts were performed using a 100 × Neofluar objective and a 10 × ocular on a minimum of 20 randomly selected fields in two replicate slides. When cell abundance was low, 30–40 random microscope fields were counted.

### Statistical Analysis

After data logarithmic transformation to comply with the assumption of normal distribution, the whole dataset including environmental parameters as well as prokaryotic abundance and microbial community metabolism was processed by analysis of variance (ANOVA) to test statistical differences occurring among the samples; a 0.05 probability level was set up as the significance threshold level. The main indices of functional diversity [i.e., substrate richness (catabolic richness, S) where the total number of wells with absorbance values over 0.10 represented the total number of oxidized carbon substrates; catabolic diversity index (Shannon functional diversity index, H); Shannon evenness (E) index] were calculated by the PAST software (version 3.14). Heatmaps of functional diversity of the microbial assemblage were also drawn using this software [27]. Pearson’s correlation coefficient analyses were calculated using the Excel software to measure the associations between pairs of parameters. To assess if there were statistical differences between groups within the multivariate dataset, the analysis of similarities (ANOSIM), a nonparametric permutation procedure that analyzes whether differences in dissimilarity between groups exceed differences within groups, was carried out by the PRIMER software, version 6.0 [28]. ANOSIM outputs produce a sample statistic, R, which represents the degree of separation between test groups: a *R* value close to 1 indicates that all replicates within a group are reciprocally more similar than any replicates of different groups, while a *R* value close to 0 indicates that there are no differences. As a post hoc test, pairwise ANOSIMs between all pairs of groups were also computed and comparisons at *P* < 0.05 were considered as statistically significant. The percentage contribution of each parameter to the observed differences between groups of samples was estimated by the SIMPER (similarity percentage) analysis [29]. Hierarchical clustering of variable data (cluster analysis) was performed by calculation of the Bray–Curtis similarity coefficients and the group average linkage method between similarities; a multi-dimensional scaling (MDS) analysis was also carried out using Bray–Curtis similarity matrices [28]. To relate the microbial data with the environmental ones, the whole dataset was also run through a principal component analysis (PCA), a multivariate analysis generating new variables, the principal components (PCs), which explain the highest dispersion of the samples. Samples were clustered according to the Euclidean distances, and the obtained information was superimposed onto the PCA plots. PCA is useful to group variables that are correlated and make predictions on processes causing these association patterns among parameters through the calculation of PC loadings, which are the correlations between each response variable and the PCs. PC loadings reveal how closely a variable and a PC are related, and the patterns of associations among variables that load on the same and/or different PCs.

## Results

### Environmental Characteristics of the Taiwan Straits

Temperature ranged between 21.53 and 25.29 °C, recorded at stations M01 and Q05, respectively. Salinity was comprised between 18.87 and 34.62 psu, measured at stations M11 and Q05, respectively (data not shown). T and S values increased moving towards offshore and from the northern transects towards the southern ones.

Total dissolved solids were in the range of 19.74–34.16 mg/L, measured at stations M11 and Q05, respectively. Significant positive relationships linked TDS distribution with S values (*r* = 0.99, *P* < 0.01). Turbidity values oscillated between − 0.85 and 9.65 FNU, observed at stations M15 and Q01, respectively. pH was comprised between a minimum of 8.01 at station XM, and a maximum of 8.27 at station M11.

The values of conductivity, salinity, and TDS measured at coastal stations were significantly lower than those at middle and offshore stations, while the values of turbidity were significantly lower (*P* < 0.05). Conversely, the concentrations of chlorophyll and PGA-PE showed no differences among the transects (*P* > 0.1).

### Microbial Community Metabolism

**Fig. 2:**
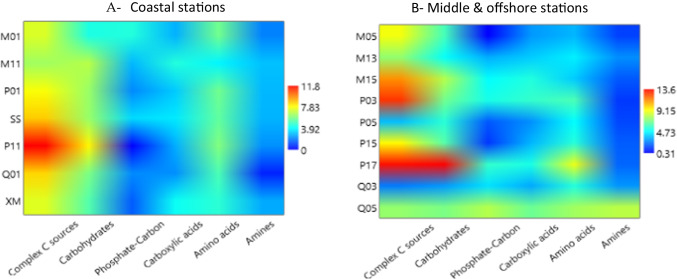
Carbon substrate utilization patterns of the microbial community observed at coastal stations compared to middle + offshore ones. Matrix plots obtained for the six guilds of metabolized substrates at coastal (**A**) and middle + offshore (**B**) stations using the interpolate option

The ASCD values measured as the absorbance values during incubation are shown in Figure S1. At all the stations, the peak values were recorded after 336 h of incubation. For further comparison of the spatial variability of microbial community metabolism, only the peak values recorded at the end of the incubation time were taken into consideration. Changes in the carbon utilization patterns over a spatial scale were evident when the coastal stations were compared to the middle + offshore ones (Fig. 2).

Globally, the lowest metabolic activity levels in terms of absorbance values were detected at station Q03, located in the middle of a southern transect, and the highest ones at station P17, an offshore station in the central zone of the study area. A decreasing coastal-offshore trend in the substrate utilization rates by the microbial community was observed, together with a general increase moving from northern to southern transects. Indeed, complex carbon sources and carbohydrates were the carbon sources preferentially metabolized, particularly in the transect P11–P17, while the lowest utilized substrates were the amines and phosphorous-carbon compounds. The lowest ability of carbon substrates utilization was detected in the transect M01–M05.

Within the metabolic profiles depicted by the Biolog Ecoplates, differences were also observed in the utilization rates of single compounds (Tables 1 and 2). Among the complex C sources, cyclodextrin and glycogen were the most frequently used substrates, while among carbohydrates, D-mannitol and N-acetyl-D-glucosamin as well as D-cellobiose were the most utilized ones. D-galactonic acid and L-asparagine were utilized substrates belonging to the guilds of carboxylic acids and amino acids, respectively.

**Table 1:**
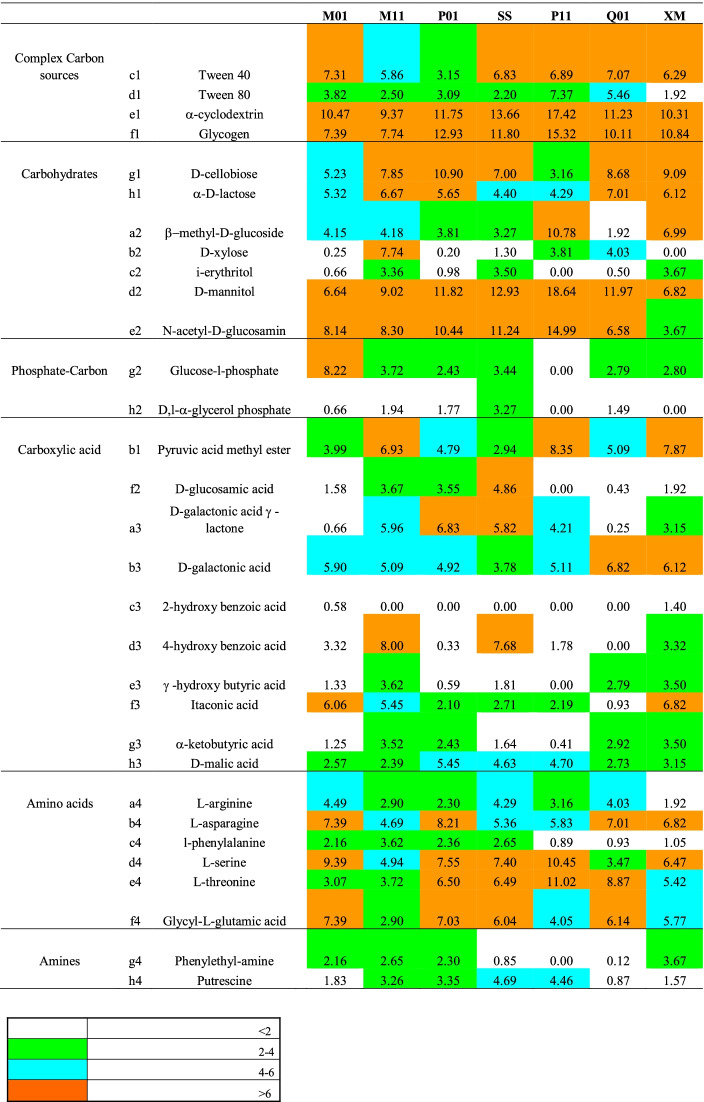
Substrate utilization patterns as optical density (OD) values recorded for each Biolog plate at the coastal stations. The legend indicates the color palette of the OD

**Table 2:**
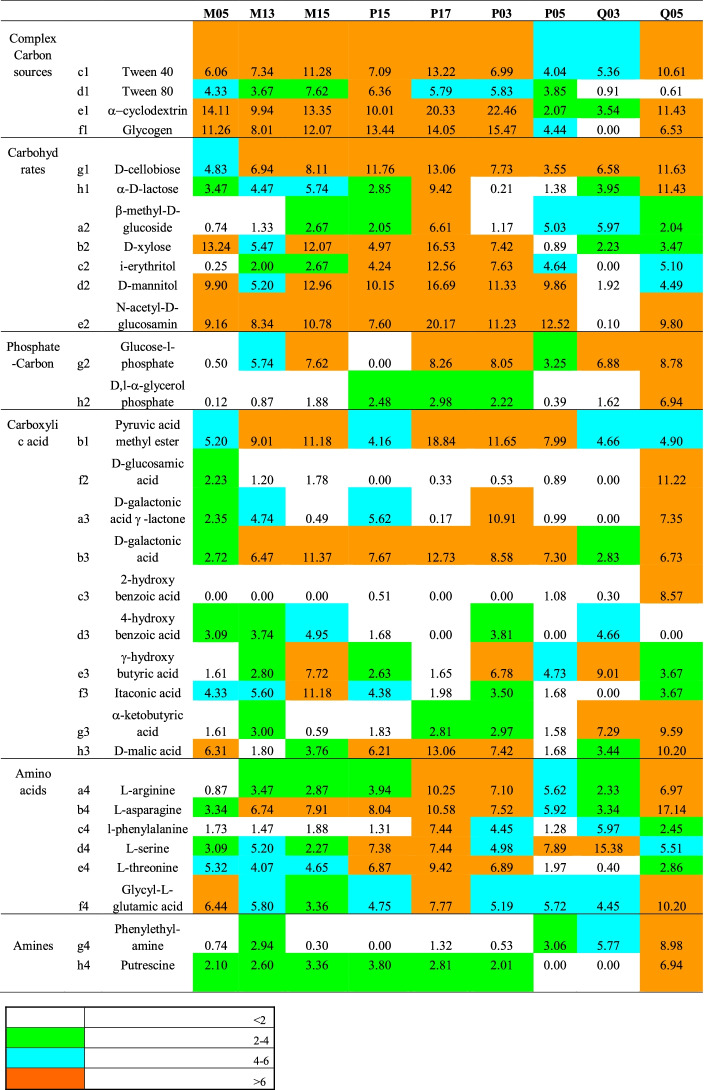
Substrate utilization patterns as optical density (OD) values recorded for each Biolog plate at the middle + offshore stations. The legend indicates the color palette of the OD

The functional diversity indices calculated on the microbial metabolic profiles are reported in Table 3. Higher numbers of oxidized substrates (S) were recorded at the northern transect M01–M05, transects M11–M13–M15, and P01–P05 as well as at station SS, compared to lower ones observed at stations P11, P15, and Q03. Similarly, the lowest substrate richness (*d*) was found at stations P11–P15 and the highest one at stations M01–M05.

**Table 3:** Diversity indices. *S*, total number of oxidized C substrates with a positive response, i.e., absorbance value over 0.10 OD; *N*, total number of positive responses per each sample; *d*, substrate richness according to the formula *d* = (*S* − 1)/Log(*N*); *J’*, Shannon functional diversity index, *H’* =  − *SUM* (Pi*Log_e_(*Pi*)), where *Pi* is the proportion of characters belonging to the *i*th type of letter and Pielou’s evenness index according to the formula *J’* = *H’*/Log(*S*) [30, 31]

Sample	*S*	*N*	*d*	*H’*(Log_e_)	*J’*
M01	31	133	6.131	3.185	0.9274
M05	30	131	5.948	3.056	0.8985
M11	30	152	5.776	3.311	0.9734
M13	30	140	5.869	3.259	0.9583
M15	30	188	5.536	3.152	0.9266
P01	30	150	5.791	3.134	0.9213
P03	30	203	5.460	3.162	0.9296
P05	29	115	5.898	3.106	0.9225
SS	30	158	5.725	3.216	0.9457
P11	24	169	4.482	2.921	0.9191
P15	28	154	5.362	3.159	0.9480
P17	29	268	5.007	3.128	0.9290
Q01	29	132	5.732	3.061	0.9091
Q03	25	109	5.117	2.952	0.9171
Q05	30	220	5.378	3.271	0.9618
XM	29	142	5.650	3.220	0.9564

Shannon functional diversity index showed quite similar values in the whole area, except for stations P11, Q01, and Q03; the same trend was observed for the Pielou’s evenness index, which is a measure of biodiversity and quantifies how equally is numerically distributed a community (in this case the number of oxidized substrates).

### Prokaryotic Abundance

**Fig. 3:**
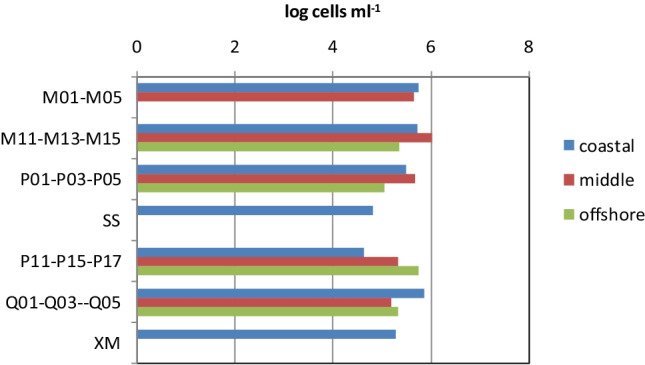
Prokaryotic abundances found at the transects studied in the Taiwan Straits

Prokaryotic abundances were in the order of 10^5^–10^6^ cells/mL, with minimum counts recorded at station P11 and the highest ones at station M13 (Table 4). Lower abundances were generally found at the offshore stations compared to those measured at the coastal ones and higher numbers were observed across the transects M and Q than across the transect P or at station SS (Fig. 3). Nevertheless, ANOVA did not show a significant variability in the spatial distribution of total prokaryotes.

**Table 4:** Prokaryotic abundances recorded at the stations sampled in the Taiwan Straits

M01	5.73 × 10^5^ cells ml^−1^
M03	4.63 × 10^5^ cells ml^−1^
M11	5.43 × 10^5^ cells ml^−1^
M13	1.09 × 10^6^ cells ml^−1^
M15	2.31 × 10^5^ cells ml^−1^
P01	3.12 × 10^5^ cells ml^−1^
P03	4.85 × 10^5^ cells ml^−1^
P05	1.15 × 10^5^ cells ml^−1^
SS	6.72 × 10^4^ cells ml^−1^
P11	4.31 × 10^4^ cells ml^−1^
P15	2.13 × 10^5^ cells ml^−1^
P17	5.71 × 10^5^ cells ml^−1^
Q01	7.27 × 10^5^ cells ml^−1^
Q03	1.59 × 10^5^ cells ml^−1^
Q05	2.17 × 10^5^ cells ml^−1^
XM	1.98 × 10^5^ cells ml^−1^

## Discussion

This study is a first contribute to knowledge of microbial distribution and function in TWS and their modulation in response to environmental gradients of nutrients and organic substrates. The extent to which organic matter at sea is processed and transformed depends on the functional capabilities of heterotrophic microbial communities [7, 32]. The carbon substrate utilization profiles obtained with the Biolog Ecoplates provided a snapshot of the actual metabolism of the microbial community in the study area; indeed, the analysis of physiological profiles using the Biolog Ecoplates allows to estimate the similarity between microbial communities from various environments and habitats. This technique has widely been applied in ecological studies of microbial communities of different wetlands [33], soils, rhizosphere, and sewage sludges or to evaluate pollution by heavy metals or hydrocarbons [34].

A patchy microbial distribution was observed in TWS, where the prokaryotic cell numbers measured in our study were in the same range of magnitude as those reported in previous investigations performed in the same geographical area. Chen et al. [35] have reported heterotrophic bacterial abundances comprised between 0.42 ± 0.13 and 4.80 ± 0.94 × 10^5^ cells ml^−1^ in the eastern coast of Taiwan between September 2012 and 2014. Jiang et al. [36] during a spring survey (May 2016) of the shallow area of the Taiwan Bank, close to the northeastern South China Sea and the southern TWS, estimated that, within the total picoplankton, the heterotrophic bacteria accounted for a concentration ranging from 9.83 ± 1.23 to 10.53 ± 1.88 × 10^5^ cells ml^−1^ in areas experiencing or not phytoplankton bloom episodes, respectively. These numbers were comparable to those observed in areas adjacent to Taiwan such as the northern South China Sea [37–39]. In the South China Sea, bacterial concentrations roughly of the same magnitude order as those in our study were also found (10^6^ cells ml^−1^) during fall 2000 and 2003 [40]. In contrast with these data, lower abundances were detected by Jiao et al. [41], in the East China during summer (July 1998), where cell concentrations ranged from 73.0 to 41.9 × 10^4^ cells ml^−1^ in coastal and offshore waters, respectively.

On a spatial scale, in our study, the highest microbial metabolic levels and prokaryotic abundance were observed in the area between Minjiang River estuary and Pingtan Island, and peaks of carbon utilization rates were found at station P17. Spatial differences in the bacterial abundances were also observed during winter in the western TWS, with significantly lower numbers in the northern area (0.4 to 1.1 × 10^6^ cells ml^−1^) than in the southern one (0.6 to 2.9 × 10^6^ cells ml^−1^) [42]. Moreover, in the northern area, a stronger bottom-up control of bacterial growth was suggested by a significant positive relationship (*r* = 0.65, *P* < 0.01) linking bacterial abundance with temperature.

The reasons for variable microbial patterns observed in our study can be likely related to the hydrological complexity of the TWS area. Here, centered on the coastal waters east of Pingtan Island and extending from the north of Pingtan Island to the south of Meizhou Island, an upwelling area with typical characteristics of low temperature, high salt, low oxygen, and high nutrients starts to appear at the end of May. The formation of this upwelling area is caused by the uplift of the terrain, the movement of the north Shanghai current, and the action of the southwest monsoon. Moreover, along the western coast of the TWS, continental runoff waters from the Oujiang, Minjiang, and Jiulongjiang rivers undergo a mixing with coastal seawater; the resulting water is characterized by low temperature and low salinity. The complex hydrological variability of the TWS is in turn reflected in the spatial heterogeneity of the microbial community metabolic profiles, suggesting the occurrence of various ecological niches and microorganisms adapted to inhabit them through diversified metabolic patterns.

The patterns of community metabolism described latitudinal and longitudinal gradients, with general trends increasing from northern to southern transects and decreasing from coastal to offshore stations. Changes in the carbon utilization patterns over a spatial scale were evident when the coastal stations were compared to the middle + offshore ones (Fig. 2); in surface waters of other marine ecosystems, the remineralization rates of dissolved organic carbon were found to be controlled by the prokaryotic community [43]. However, in the TWS, there were no significant differences among the transects in the concentration of chlorophyll-*a* and prokaryotic abundance. Therefore, the role of prokaryotes in the water carbon cycle did not depend on their abundance but rather on their composition. Driven by the coastal water mass of Zhejiang and Fujian [14, 15], in this survey, the coastal waters showed a significantly lower salinity than offshore waters, where prokaryotes had strong carbon hydrolysis capacities. In addition, the relationship between organisms also could be affected by the salinity, thereby influencing the utilization of organic matter in the water bodies [44]. In the East China Sea, phytoplankton bloom mainly occurred between May and July and the highest cell densities were found in the coastal waters [45]. So, we hypothesized that the contribution of prokaryotes to the hydrolysis of organic carbon could provide inorganic nutrients to support the following algal blooms along the coast.

Heterotrophic bacteria usually uptake directly low molecular weight compounds only (i.e., amino acids, simple sugars, and fatty acids), while high molecular weight polymers require to be hydrolyzed into small, simpler monomers prior their uptake and incorporation into new microbial biomass [8]. Moreover, the spatial and temporal distribution and metabolic patterns of the microbial community often reflect the variability in the nature and concentration of organic polymers, as well as of microbial community composition [9, 33].

Although different utilization patterns of the different carbon source guilds occurred at each station, among the assayed organic substrates, the microbial community present in TWS was found to metabolize mostly complex carbon sources, followed by carbohydrates, particularly in the transects P11–P17. The microbial capacity for using C sources was likely related to the presence of compounds derived from the autochthonous primary production.

Complex carbon sources such as the polyols were efficiently utilized everywhere; some of these compounds are recognized to be emulsifiers, surfactants, and solubilizers, like Tween 40, Tween 80, and α-ciclodextrin, or to act as energy reserve like glycogen. A preference for the utilization of Tween 40, Tween 80, and γ-hydroxybutyric acids, all fatty acids degraded by microbes able to use lipids, was observed in the microbial community inhabiting the water compartment of aquaculture ponds [46] found that putrescine, Tween 80, L-serine, D, L-a-glycerol, α-ketobutyric acid, D-mannitol, D-cellobiose, D-galactonic acid γ-lactone, and L-arginine were the main carbon sources used by microbial communities in aquaculture pond waters. N-acetyl-D-glucosamine is one of the monomers of chitin, a major component of the crustaceans exoskeleton.

Carbohydrates were found to be actively metabolized by the prokaryotic community also in other studies [7, 9, 32]. Simple carbohydrates, such as glucose, are the most common and easily metabolized form of organic C by microorganisms. D-cellobiose, being a product of cellulose hydrolysis, could indicate the occurrence of allochthonous organic carbon of algal origin; D-cellobiose comes from cellulose hydrolysate; this substrate could be related to phytoplankton biomass [47]. Both carbohydrates and phosphate-carbon sources may represent an energy reservoir. Among the phosphate-carbon compounds, glucose-1-phosphate was mostly used. The carboxylic acids are constituents of hydrophobic biomolecules. Among these substrates, the pyruvic acid could be utilized in the methanogenesis. This compound derives from pyruvic acid methyl ester and is a product of glycolysis. Esters of pyruvic acid support cell chemical activity; the pyruvic acid methyl ester is used by a variety of environmental bacterial communities [47].

Amino acids are the product of decomposition of proteins that are the most frequent substrates found at sea [7, 9, 32]; the results showed that, among them, compounds such as L-arginine and L-asparagine represent a common source of carbon, nitrogen, and energy, which is derived mainly from primary production. L-phenylalanine is an amino acid used in the synthesis of flavonoids, which are involved in UV filtration, symbiotic nitrogen fixation, and pigmentation. Low microbial utilization of amines was observed in TWS.

### Microbial Response to Environmental Parameters and Functional Associations

Pearson’s correlation coefficients calculated between the metabolic profiles and the environmental parameters showed positive relationships between the microbial utilization ability of amino acids and T (*r* = 0.676, *P* < 0.01) as well as S (*r* = 0.580, *P* < 0.01). No correlations were found between the prokaryotic abundance and the microbial capability of using C substrates; a similar lack of relationships between prokaryotic abundances and metabolic profiles, reported by Bullock et al. [48] in two temperate coastal rivers of North Carolina, was referred to depend on the kinetic characteristics of microbial enzymes and their active lifetimes.

Positive relationships were observed in our study between complex C sources and carbohydrates (*r* = 0.743, *P* < 0.01) as well as between amino acids and carbohydrates (*r* = 0.592, *P* < 0.01) or phosphate-carbon sources (*r* = 0.560, *P* < 0.01). Amines and phosphate-carbon sources were reciprocally related too (*r* = 0.561, *P* < 0.01), as well as amines and carboxylic acids (*r* = 0.575, *P* < 0.01) (data not shown in the figure). Comparing coastal *versus* middle + offshore stations, an increase in the number of substrates used at an absorbance threshold > 6 was observed; nevertheless, on a spatial scale, ANOVA did not point out significant differences in the microbial community metabolism with respect to the six guilds of assayed substrates. The outputs of ANOSIM analysis highlighted the difference between coastal and offshore stations, with an average squared distance of 72.93 between coastal and offshore stations, 47.02 between coastal and middle stations, and 81.70 between middle and offshore stations. SIMPER analysis showed that the main parameters contributing to such differences were lactose and acetylglucosamine at coastal stations, while mannitol, hydroxybutyric acid, and Tween 80 showed a predominant role at offshore stations. MDS analysis revealed a close association among the ability to use complex carbon sources and carbohydrates, while carboxylic acids grouped together with amines and phosphate-carbon compounds. Cluster analysis among stations revealed the occurrence of three main sub-systems: cluster 1, including stations P01, P11, P15, SS, P5, and Q03; cluster 2, including stations M01–M05 and M13; and cluster 3, with stations P03 and M15 (data not shown in the figure).

**Fig. 4:**
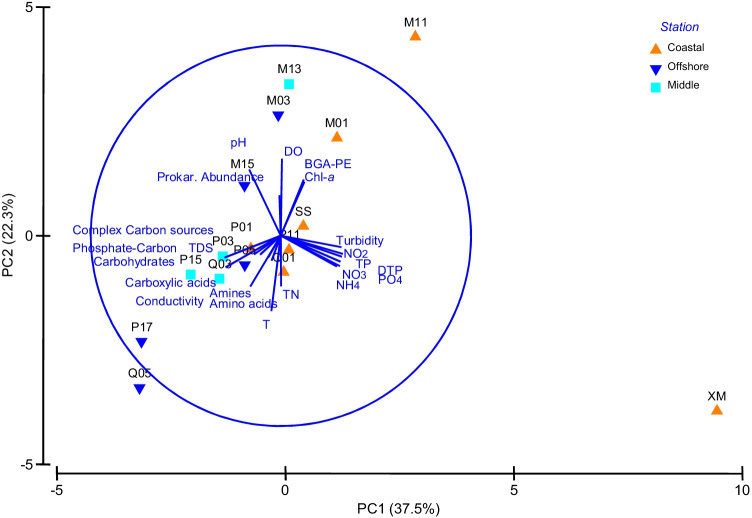
Results of Principal Component Analysis performed on the microbial and environmental dataset. Abbreviations: T, temperature; DO, dissolved oxygen; TP, total phosphorus; DTP, dissolved total phosphorus; PO_4_, ortophosphate; TN, total nitrogen; NH_4_, ammonia; NO_2_, nitrite; NO_3_, nitrate; TDS, total dissolved solids; Chl-*a*, chlorophyll-a; BGA-PE, blue-green algae-phycoerythrin

PCA calculated on microbial and environmental variables, reported in Fig. 4, showed that PC1 and PC2 justified 37.5% and 22.3% of the total variance, respectively. Both the PCs were a combination of variables, with a greater weight for NO_2_, NO_3_, TP, and PO_4_, S and TDS on PC1. Chl-*a*, BGA-PE, and PA were positively related.

PC2 was dominated by dissolved oxygen, pH, and T. Negative correlation coefficients were found between the prokaryotic abundance, nutrients, and turbidity, as well as between all the substrate types and Chl-*a* and BGA-PE, suggesting that carbon substrates fuelled microbial metabolism.

## Conclusions

A snapshot of the microbial abundance and activity in TWS as a model site of aquatic ecosystems impacted from land inputs has been obtained. The metabolic functions of the TWS waters appeared to be quite diversified. A patchy microbial distribution was generally observed, with the highest microbial metabolic levels and prokaryotic abundance in the area between Minjiang River estuary and Pingtan Island, and progressive decreases towards offshore stations. Complex carbon sources and carbohydrates were preferentially metabolized.

The obtained data highlights that microbial community is significantly affected by environmental conditions such as the presence of coastal to offshore gradients and that microbial metabolism is more sensitive than abundance to environmental changes.

## Supplementary Information

Below is the link to the electronic supplementary material.

Supplementary file1 **Fig. S1** Average Substrate Color Development (ASCD) over time recorded at each station sampled in the Taiwan Straits (DOCX 92 KB)

## Data Availability

The datasets generated during and/or analyzed during the current study are available from the corresponding author on reasonable request.
